# How does COVID-19 fear and anxiety affect chemotherapy adherence in patients with cancer

**DOI:** 10.2217/fon-2020-0592

**Published:** 2020-07-17

**Authors:** Cengiz Karacin, Irem Bilgetekin, Fatma B Basal, Omur B Oksuzoglu

**Affiliations:** ^1^Department of Medical Oncology, HSU Dr Abdurrahman Yurtaslan Oncology Training & Research Hospital, Ankara 06200, Turkey

**Keywords:** anxiety, cancer, chemotherapy adherence, COVID-19, fear, telemedicine

## Abstract

**Aim:** To investigate how COVID-19 fear and anxiety (COV-FA) affects chemotherapy adherence in patients with cancer. **Materials & methods:** The records of 3661 patients with chemotherapy (CT) appointments were retrospectively reviewed. **Results:** The CT postponement rates before and after COVID-19 were 11.6% and 14.2%, respectively (p = 0.017). The rate of COV-FA-related CT postponement after telemedicine was lower than that before (4.6% vs 17.4%, p = 0.012). The median time to come back to treatment of the COV-FA group was 47 days (range 19–72 days). Advanced age (≥60 years) was found to be the independent factor that was predictive of time to come back to treatment (p = 0.043). **Conclusion:** The CT postponement rate increased after COVID-19. COV-FA-related CT postponement decreased after telemedicine. Advanced age could be predictive of time to come back to treatment.

Systemic treatments, especially cytotoxic chemotherapy (CT), play an important role in the treatment of cancer [1,2]. The main goal in the treatment of localized diseases is to eliminate the disease if possible and to prevent recurrence, while the aim in treating advanced-stage disease is to control the disease [1,2]. To prevent recurrence of the disease or to control the disease, adjuvant or palliative CT is administered in many types of cancer [1,2]. Implementation of planned CT without delay is extremely important for treatment effectiveness [3]. Studies have shown that postponing CT for any reason negatively affects the survival of patients [3]; therefore patient adherence to treatment directly affects the treatment response [3]. Psychological disorders such as anxiety and depression are a factor that adversely affects patient adherence to CT [4,5].

Starting in December 2019 in Wuhan, China, the new coronavirus disease (COVID-19) quickly spread all over the world and was declared as the COVID-19 pandemic by the World Health Organization on 11 March 2020 [6]. Rapid spread of the disease and its high mortality caused fear and anxiety among people [7]. With the ‘social distancing’ and ‘stay home’ strategies implemented by governments, the rate of the spread of the disease diminished in some regions [8]. Studies have shown that the prevalence of anxiety and other psychological disorders increased during the COVID-19 pandemic [7]. COVID-19-related fear and anxiety (COV-FA) escalated because of the lockdown, the lack of a treatment or vaccine to eradicate the disease and the lack of estimation on when the pandemic will end [7,8].

Immunosuppression in patients with cancer, caused both by their disease and by the therapies they have received, makes them more vulnerable during the COVID-19 pandemic [9]. In fact, in the retrospective studies published, COVID-19 mortality was reported to be higher in patients with cancer [9]. Patients are exposed to news reports stating that mortality is higher in people with chronic diseases, which can raise concerns [10]. Some patients who think hospitals pose a risk for disease transmission are thought to postpone their follow-up visits or treatments due to COV-FA [7]. As far as we know, a study of how COV-FA affects the treatment process for patients with cancer, who are receiving active CT, has not yet been published. The present study is therefore intended to investigate CT adherence and CT postponement rates, and their relationship with COV-FA in patients receiving active CT for cancer, during the COVID-19 pandemic. The study also intends to assess time to come back to treatment (TCBT) in patients who postponed their CT due to COV-FA and to identify the factors affecting TCBT.

## Materials & methods

### Patients & study design

Patients with appointments between 17 January 2020 and 10 May 2020 were retrospectively reviewed on the appointment list of patients who were going to receive CT at our healthcare center. Patients who did not come for CT on the date of their appointment were identified by reviewing the patient records. The CT postponement rates were calculated for the 60-day periods before and after 10 March 2020, when the first COVID-19 case was diagnosed in Turkey. We planned to include all adult patients (>18 years of age) with postponed CT and histopathologically diagnosed cancer. Illiterate patients, those with symptomatic brain metastases that could disrupt cognitive functions, those with psychiatric disorders and those with anxiety disorders were excluded from the study. To obtain the most accurate information about the psychiatric and anxiety disorders of the patients, we made a two-step evaluation. Firstly, we examined the follow-up notes and histories in our patient follow-up forms in detail and hospital patient electronic data file were retrospectively screened for any information about psychiatric problems. Secondly, we asked each patient whether they had a history of psychiatric or anxiety disorder.

Data on patients’ age, sex, comorbidities, history of smoking, marital status, number of children, educational background, place of residence and household, cancer type, disease stage, CT regimen and date of CT postponement were obtained from the hospital records. A patient questionnaire (prepared using the Google survey program) that involved the Beck anxiety inventory (BAI) and questions about COVID-19 fear was sent to patients on WhatsApp, along with the informed consent form. A database was created using the data obtained from the hospital records and the patients’ responses in the questionnaire.

### Identification of the causes of CT postponement & the formation of patient groups

The reasons given by the patients who postponed CT were first investigated using the hospital records. Patients who reported that they did not come to their CT appointment due to COV-FA according to the hospital records were called by phone to confirm their reason for CT postponement and the COV-FA group was formed. Patients who had postponed their CT but had no record of the reason for the postponement in the hospital records were called; those who stated they postponed their CT due to COV-FA were also included in the COV-FA group. During the telephone conversations with patients in the COV-FA group, the patients were invited to participate in the survey study and all of these patients (30 patients) agreed. Those with postponed CT due to neutropenia, thrombocytopenia, acute renal failure, drug-related side effects like skin toxicity and non-COV-FA reasons such as infection and thrombosis were also invited to participate in the survey study and formed the ‘other’ group (80 patients). The demographic and clinical characteristics, questionnaire responses and BAI scores of both groups (30 patients in the ‘COV-FA’ and 80 patients in the ‘other’ group) were compared. Factors associated with CT postponement due to COV-FA were identified.

### Telemedicine application

We started the telemedicine practice 35 days after the first COVID-19 case in Turkey for patients who had been receiving treatment at the Medical Oncology Chemotherapy Day Unit. As part of this practice, patients with a booked CT appointment were called by a medical oncology specialist doctor a few days before their appointment. During these phone calls, patients were asked whether they had any symptoms of COVID-19 (fever, cough, sputum and shortness of breath), whether they had been in contact with someone infected with COVID-19 and whether they had traveled abroad in the last 14 days. The patients were also informed about whether there were COVID-19 cases in our healthcare center as of the day the phone call was made. Any questions the patients asked about COVID-19 were answered.

### Follow-up of the COV-FA group & termination of the study

The hospital records of the patients who postponed their CT appointments due to COV-FA on the day of freezing the database for the present study were reviewed again and a record of whether they had returned to our clinic to receive CT again was added to the database. The dates when patients returned to receive CT again were recorded. Those who had not yet been readmitted for CT administration were interviewed by phone and the last contact date for these patients was recorded as May 28, 2020, the date of freezing the study database.

### Beck Anxiety Inventory

The BAI is a multiple-choice self-report inventory used for measuring the severity of anxiety in children and adults [11]. It was developed by Beck *et al.* and a study of its reliability and validity in Turkey was carried out by Ulusoy *et al.* [12] The questions used in this measure ask about common symptoms of anxiety that the subject has had during the past week (including the day on which the test is taken), such as numbness and tingling, sweating not due to heat and fear of the worst happening [11]. The BAI contains 21 questions, each answer being scored on a scale value of 0 (not at all) to 3 (severely) [11]. Higher total scores indicate more severe anxiety symptoms [11].

### Statistical analysis

Data analysis was performed using IBM SSPS Statistics for Windows v.20.0 software (IBM, NY, USA). Qualitative variables were expressed as frequencies and percentage, whereas the quantitative variables were expressed as mean (± standard deviation) or median. The conformity of the numerical data to the normal distribution was assessed using the Kolmogorov–Smirnov test. The Student *t* test was used to compare the parametric data and the Mann–Whitney *U* test was used to compare the nonparametric data. Pearson's chi-square or Fisher's exact test was performed to compare the categorical data. TCBT was defined as the elapsed time from CT postponement until either return for treatment or the last contact. TCBT was determined using the Kaplan–Meier method and the log-rank test was used for univariate comparison. A multivariate Cox regression model was used to identify independent predictive factors on TCBT. All statistical analyses were two-way and the level of statistical significance was set at p < 0.05.

## Results

**Figure 1.**
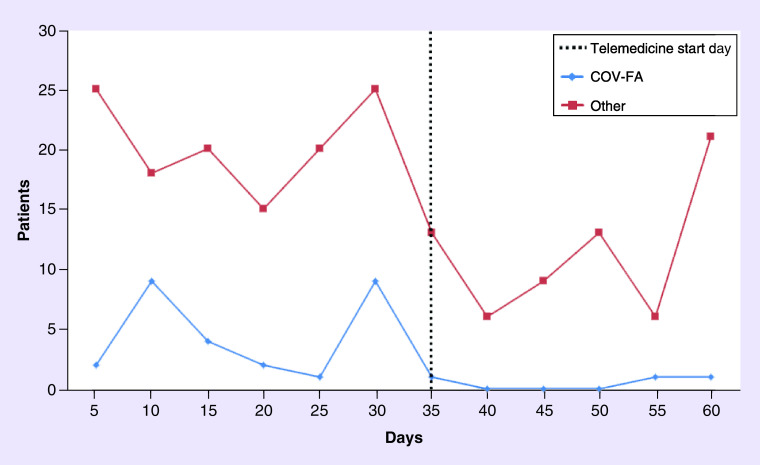
Distribution of patients with cytotoxic chemotherapy postponement before and after telemedicine by reasons for cytotoxic chemotherapy p **ostponement.** COV-FA: COVID-19 fear and anxiety.

Of 2112 patients with CT appointments in the 60-day period preceding the first COVID-19 case (10 March 2020), 245 (11.6%) had their CT appointment postponed; of a total of 1549 patients with a CT appointment in the 60-day period following the first COVID-19 case, 220 (14.2%) had their CT appointment postponed (χ^2^ = 5.729, p = 0.017). The three most common reasons for postponing CT after COVID-19 were neutropenia (23.1%), thrombocytopenia (20.9%) and COV-FA (13.6%; Table 1); 27 patients (17.4%) postponed CT due to COV-FA before the introduction of the telemedicine practice, but only three patients (4.6%) postponed CT for this reason (p = 0.012; Figure 1) after the telemedicine practice was initiated.

**Table 1.** Reasons for postponement of chemotherapy after the first COVID-19 case.^†^

Characteristic	n (%)
Neutropenia	51 (23.1)
Thrombocytopenia	46 (20.9)
Fear and anxiety	30 (13.6)
Infection	17 (7.7)
Progressive disease	12 (5.5)
ECOG PS	9 (4.1)
Transportation problem	9 (4.1)
Anemia	5 (2.3)
Acute thrombosis	5 (2.3)
Metabolic	5 (2.3)
Other	31 (14.1)

^†^ n = 220

ECOG: Eastern Cooperative Oncology Group; PS: Performance status.

The median age of the 110 patients who participated in the study was 63 years (range 54–70); 36.4% were male and 63.6% were female. The demographic and clinical characteristics of all patients in the COV-FA group and the other group are shown in Table 2. The percentage of female patients in the COV-FA group was significantly higher than that in the other group (80.0 vs 57.5%, p = 0.049). There was no significant difference between the two groups in terms of age, disease stage or CT administration modality.

**Table 2.** Comparison of the demographic and clinical features of the COV-FA and other groups.

Characteristic	Total (n = 110)	COV-FA (n = 30)	Other (n = 80)	p-value
Age, year, median (IQR)	63 (54–70)	63 (52–71)	63 (54–68)	0.631
Gender, n (%)				
– Male	40 (36.4)	6 (20.0)	34 (42.5)	**0.049**
– Female	70 (63.6)	24 (80.0)	46 (57.5)	
Cancer, n (%)				
– Breast	28 (25.5)	13 (43.3)	15 (18.8)	NA
– Colorectal	22 (20.0)	7 (23.3)	15 (18.8)	
– Ovarian	12 (10.9)	3 (10.0)	9 (11.2)	
– Lung	11 (10.0)	2 (6.7)	9 (11.2)	
– Pancreas	9 (8.2)	2 (6.7)	7 (8.8)	
– Gastric	8 (7.3)	0 (0)	8 (10.0)	
– Other	20 (18.1)	3 (10.0)	17 (21.2)	
Stage, n (%)				
– II	10 (9.1)	3 (10.0)	7 (8.8)	0.323
– III	23 (20.9)	9 (30.0)	14 (17.5)	
– IV	77 (70.0)	18 (60.0)	59 (73.8)	
Regimen, n (%)				
– Platin doublet	25 (22.7)	7 (23.3)	18 (22.4)	NA
– Paclitaxel	11 (10.0)	4 (13.3)	7 (8.8)	
– FOLFOX-6	9 (8.2)	2 (6.7)	7 (8.8)	
– Gemcitabine	7 (6.4)	0 (0)	7 (8.8)	
– Capecitabine + oxaliplatin	6 (5.5)	1 (3.3)	5 (6.2)	
– 5-FU based + Anti-EGFR	6 (5.5)	2 (6.7)	4 (5.0)	
– 5-FU based + Anti-VEGF	6 (5.5)	2 (6.7)	4 (5.0)	
– FOLFIRI	4 (3.6)	0 (0)	4 (5.0)	
– Other	36 (32.6)	12 (40.0)	24 (30.0)	
CT, n (%)				
– Neoadjuvant	5 (4.5)	1 (3.3)	4 (5.0)	0.583
– Definitive	2 (1.8)	1 (3.3)	1 (1.2)	
– Adjuvant	25 (22.7)	9 (30.0)	16 (20.0)	
– Palliative	78 (70.9)	19 (63.4)	59 (73.8)	

COV-FA: COVID-19 fear and anxiety; CT: Chemotherapy; IQR: Interquartile range; NA: Not applicable; FOLFIRI: Folinic acid, fluorouracil and irinotecan.

In total, 110 patients who responded to the questionnaire, including those with COV-FA-related CT postponement or CT postponement due to other reasons, were compared in terms of their social characteristics, survey results and BAI. The groups were similar in terms of marital status, place of residence, educational background, comorbidity, history of smoking and access to the hospital. The COV-FA group had a higher rate of ‘Yes’ responses to the questions ‘Are you afraid of COVID-19?’, ‘Does it bother you to think about COVID-19?’, ‘Does the concern of getting COVID-19 disrupt your sleep?’, ‘Do you worry about COVID-19 news and stories you read on social media?’, ‘Does the thought of getting COVID-19 give you palpitations?’ than the other group (Table 3). The mean BAI of the COV-FA group was significantly higher than that of the other group (18.9 vs 3.3, p < 0.001).

**Table 3.** Comparison of COV-FA and Other groups among the surveyed patients.

Characteristic	COV-FA (n = 30)	Other (n = 80)	p-value
Marital status, n (%)			
– Married	25 (83.3)	72 (90.0)	0.312
– Single	4 (13.4)	4 (5.0)	
– Divorced	1 (3.3)	4 (5.0)	
Place of residence, n (%)			
– Urban	25 (83.3)	69 (86.2)	0.763
– Rural	5 (16.7)	11 (13.8)	
Education level, n (%)			
– Primary school	18 (60.0)	57 (71.2)	0.258
– Middle-High school	10 (33.3)	15 (18.8)	
– University	2 (6.7)	8 (10.0)	
Living with, n (%)			
– Family	14 (46.7)	24 (30.0)	0.351
– Spouse	8 (26.7)	23 (28.8)	
– Child	7 (23.3)	26 (32.4)	
– Nobody	1 (3.3)	7 (8.8)	
Transportation, n (%)			
– Own	20 (66.7)	62 (77.5)	0.245
– Public	10 (33.3)	18 (22.5)	
Comorbidity, n (%)			
Yes	15 (50.0)	24 (30.0)	0.051
No	15 (50.0)	56 (70.0)	
Smoking, n (%)			
– Yes	5 (16.7)	13 (16.5)	0.873
– Quit	8 (26.7)	25 (31.6)	
– Never	17 (56.7)	41 (51.9)	
Are you afraid of COVID-19? Yes, n (%)	29 (96.7)	27 (33.8)	<0.001
Does it bother you to think about COVID-19? Yes, n (%)	26 (86.7)	26 (32.5)	<0.001
Do you think COVID-19 may affect you differently than other people because of your cancer? Yes, n (%)	21 (70.0)	55 (68.8)	0.954
Do you follow the news about COVID-19 and stories on social media? Yes, n (%)	23 (76.7)	68 (85.0)	0.303
Do you worry about COVID-19 news and stories you read on social media? Yes, n (%)	20 (66.7)	28 (35.0)	0.003
Does the concern of getting COVID-19 disrupt your sleep? Yes, n (%)	6 (20.0)	0 (0)	<0.001
Does the thought of getting COVID-19 give you palpitations? Yes, n (%)	5 (16.7)	0 (0)	0.001
Do you have any relatives who have had COVID-19? Yes, n (%)	0 (0)	0 (0)	NA
Is anybody over 60 years old living with you? Yes, n (%)	14 (46.7)	38 (47.5)	0.938
Beck anxiety score, mean ± sd	18.9 ± 9.4	3.3 ± 1.6	<0.001

COV-FA: COVID-19 fear and anxiety; NA: Not applicable.

**Figure 2.**
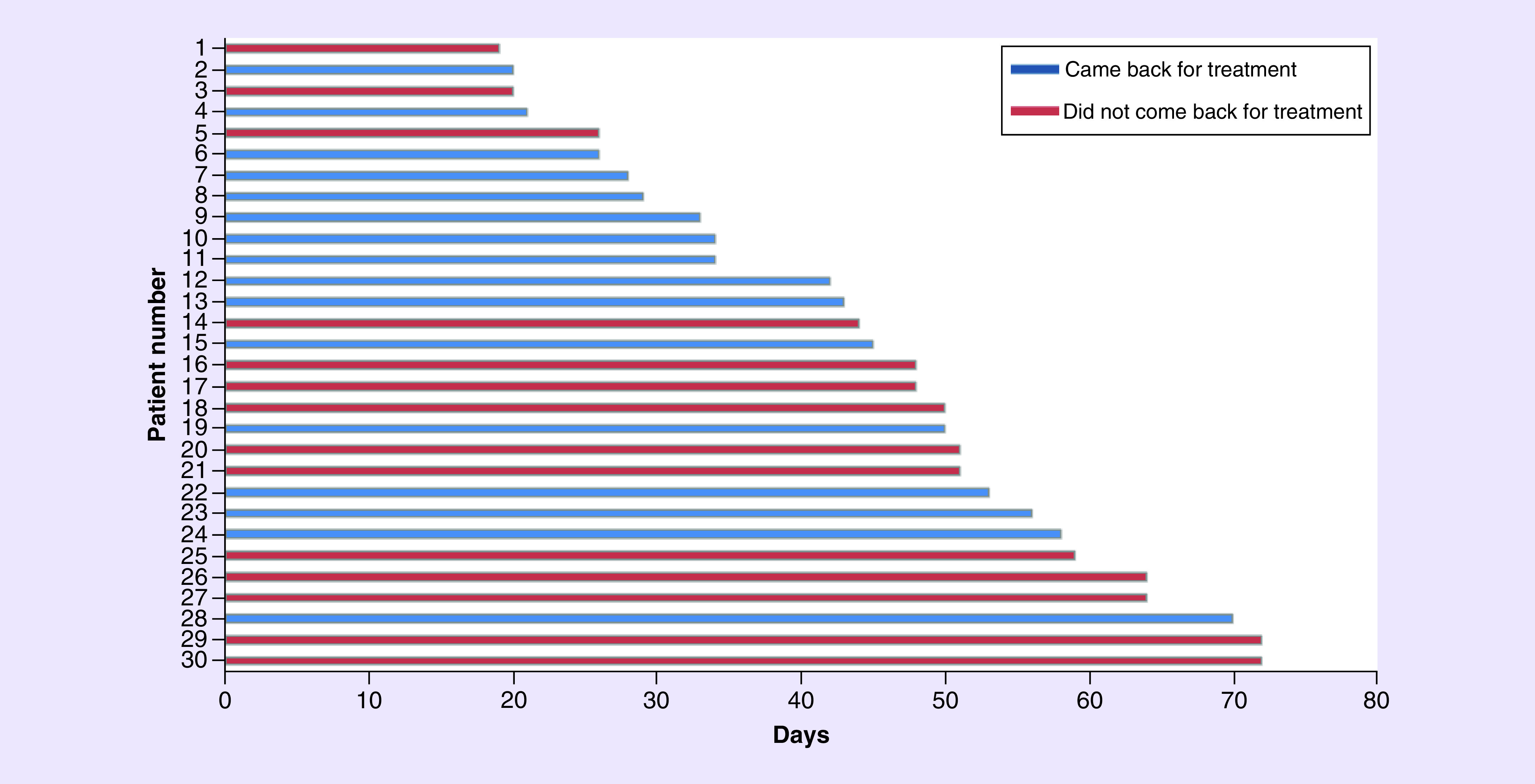
The duration of postponed chemotherapy.

As of the date when the study analyses were performed, 16 of the 30 patients in the COV-FA group returned to receive CT again, while 14 were not admitted by our clinic for CT again. We contacted those 14 patients and they stated they did not want to return to their treatment for a while due to the pandemic process. The median CT postponement time of the patients in the COV-FA group was 47 days (range 19–72; Figure 2).

**Figure 3.**
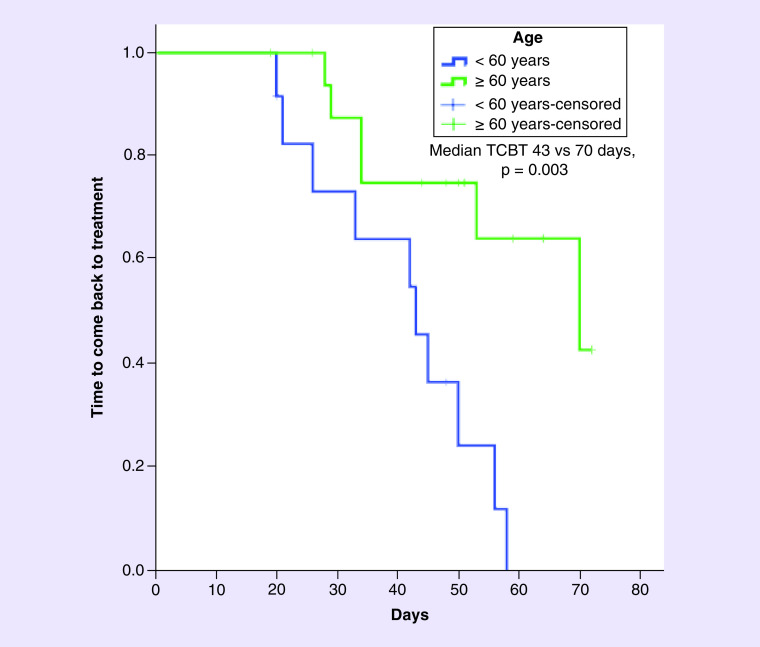
Time to come back to treatment according to age.

Following univariate analysis of the factors that may affect the TCBT of the patients, advanced age (≥60 years) and advanced-stage disease were associated with longer TCBT. The median TCBT of patients with stage II, III and IV disease were 33, 53 and 70 days, respectively (p = 0.048; Table 4). The median TCBT was found to be longer in those aged ≥60 years than in those aged <60 years (70 vs 43 days, p = 0.003; Figure 3). The median TCBT of those receiving palliative treatment was 70 days and the mean for those who did not receive palliative treatment was 45 days (p = 0.072).

**Table 4.** Univariate analysis of the factors affecting TCBT.

Characteristic	Median TCBT, days (95% CI)	p-value
Age, n (%)		
– <60	43 (38.2–47.8)	**0.003**
– ≥60	70 (39.4–100.6)	
Gender, n (%)		
– Male	34 (31.9–36.1)	0.623
– Female	56 (46.2–65.8)	
Marital status, n (%)		
– Married	58 (34.3–81.7)	0.518
– Single	50 (17.1–82.9)	
– Divorced	45 (NA)	
Place of residence, n (%)		
– Urban	53 (44.4–61.6)	0.746
– Rural	34 (31.9–36.1)	
Education level, n (%)		
– Primary school	70 (34.9–105.1)	0.285
– Middle-high school and University	50 (38.3–61.7)	
Comorbidity, n (%)		
– Yes	50 (22.3–77.7)	0.570
– No	53 (39.3–66.7)	
Smoking, n (%)		
– Yes	45 (NA)	0.982
– Quit	50 (8.9–91.1)	
– Never	56 (41.1–70.9)	
Stage, n (%)		
– II	33 (13.8–52.2)	**0.048**
– III	53 (37.1–68.9)	
– IV	70 (49.4–90.6)	
Treatment, n (%)		
– Palliative	70 (49.5–90.5)	0.072
– Other	45 (28.1–61.9)	
Beck anxiety score, n (%)		
– 0–15	NR	0.418
– >15	53 (33.5–72.5)	

NR: Not reached; TCBT: Time to come back to treatment.

As a result of the univariate analysis, a Cox regression model was created based on the factors whose p-value was <0.20 (age, stage and treatment modality; Table 5). As a result of the multivariate analysis, advanced age (≥60 years) was found to be an independent factor predicting TCBT (hazard ratio = 0.291, p = 0.043).

**Table 5.** Multivariate Cox regression analysis of the factors affecting TCBT.

Characteristic	HR (95% CI)	p-value
Age		
– <60	Reference	0.043
– ≥60	0.291 (0.088–0.963)	
Stage		
– II	Reference	0.787
– III	0.595 (0.136–2.611)	
– IV	NA	
Treatment		
– Palliative	Reference	0.941
– Other	NA	

HR: Hazard ratio; NA: Not applicable; TCBT: Time to come back to treatment.

## Discussion

As far as we know, ours is the first study to investigate COV-FA within the context of CT adherence in patients with cancer receiving active CT. In the present study, it was seen that the CT postponement rate increased significantly following the first COVID-19 case in Turkey and COV-FA was identified as the third most frequent reason for CT postponement. COV-FA-related CT postponement was more frequent in women than in men. Patient age was also found to be an independent factor that predicted TCBT. Patients aged ≥60 years had longer TCBT than those aged <60 years.

Studies have shown that anxiety disorders have negative effects on adherence to CT in patients with cancer [4,5]. In their study involving 135 patients with colon cancer, Zhu *et al.* investigated the causes that led to postponement of adjuvant CT initiation; they concluded that anxiety is an independent predictive factor for postponing CT [5]. In a prospective study that included 50 patients with advanced lung cancer, Greer *et al.* showed that anxiety is one of the factors that negatively affects adherence to chemotherapy [4]. The present study also investigates how elevated anxiety and fear in society during the pandemic can affect the chemotherapy process. The significant difference found in the CT postponement rates in our oncology center before and after the first COVID-19 case (11.6 vs 14.2%) suggested that it could be due to COV-FA. COV-FA was the third most common cause among the reasons for postponing CT after the first COVID-19 case. Patients who have had COVID-19 infection may be more likely to develop fear and anxiety; however, because there were no COVID-19 infected patients in our study, we could not comment on CT postponement rates in these patients.

The present study revealed that female sex can be a risk factor for COV-FA, which is consistent with the literature. Many studies have proven that anxiety disorders occur more often in women [13]. In the study conducted by Braamse *et al.* to investigate factors associated with the causes of anxiety and depression in patients with colorectal cancer, women were twice as likely as men to have anxiety [14]. The relationship between anxiety and female sex has been found to be similar in studies of the COVID-19 pandemic, including one in Turkey [7,15]. A meta-analysis showed that COVID-19 anxiety was particularly more common in female healthcare workers than in male healthcare workers [15]. One study showed that chronic diseases and living in the city center also increase the risk of anxiety [13]. In the present study, there was no significant difference between the patients with CT postponement due to COV-FA and those with CT postponement due to other reasons in terms of chronic diseases and place of residence.

Telemedicine provides an important advantage in protecting patients with cancer from COVID-19 infection because these patients already have immunosuppression [16]; 35 days after the first COVID-19 case in Turkey, we started interviewing follow-up patients at our oncology center using telemedicine. Patients’ follow-up, examination results and treatment responses were all evaluated during video calls. Moreover, the patients were questioned about whether they had symptoms of COVID-19, thereby ensuring the early diagnosis of a possible COVID-19 infection and reducing the risk of these patients infecting other patients in the CT day unit. During the interviews, we shared up-to-date information about whether COVID-19 cases were seen in our healthcare center and encouraged patients to continue their treatment by informing them about the undesired consequences of treatment discontinuation. These interviews using telemedicine might have yielded positive results, especially in terms of CT postponement rates. CT postponement rates related to COV-FA decreased after the telemedicine application had started; however, it would not be correct to associate this decrease in COV-FA-related CT postponement rate to telemedicine alone.

We have not encountered any study in the literature that examines the TCBT in patients with postponed CT. In the present study, the median TCBT of the patients who postponed CT due to COV-FA was 47 days (range 19–72). The multivariate analysis showed that only advanced age is an independent factor that predicts TCBT. Studies have shown that advanced age is an important prognostic factor in terms of COVID-19 mortality and this information has been frequently covered in the news and social media [17,18]. We believe that the long TCBT of the elderly patients herein may be related to the coverage of this information in the news. As a matter of fact, one of the questions the patients were asked as part of the present study was whether they were following news or social media concerning COVID-19; the majority (76.7%) of the patients who postponed their CT due to COV-FA responded ‘Yes’ to this question.

Repeated media exposure to community crisis can lead to increased anxiety and studies have shown that media and social media exposure might increase COVID-19 anxiety [19,20]. People have been exposed to news of COVID-19 in different ways and have different ideas of the severity of the pandemic. Our study did not measure media exposure or social media exposure by an objective method; however, rates of following the news or social media concerning COVID-19 were similar between the ‘COV-FA’ and ‘other’ groups in our study. Rural residents may have lower access to and use of certain health information sources relative to urban residents [21]. Considering that the difference between rural and urban residents might affect COVID-19 anxiety, we compared the ‘COV-FA’ and ‘other’ groups in terms of the regions where the patients live but did not find any difference between the groups.

The present study has some limitations. Patients included in the present study were very heterogeneous in terms of their cancer diagnoses and only a small number of patients were included. Another limitation arising from the retrospective design of the study is that the elapsed time from the date of survey delivery to the patients and the date of CT postponement was not the same for all patients. Patients with psychiatric disorders were excluded from the study; however, cancer-related anxiety that we have not yet noticed (especially in patients at the beginning of the diagnosis and treatment process) might be confounding some of the results. Our study was conducted within a short period of time after the first COVID-19 case. It would be useful to support our findings using longitudinal studies to generalize our results, because different results can be obtained with long-term studies.

## Conclusions

The present study concludes that COV-FA increases CT postponement rates, telemedicine can contribute to reducing COV-FA-related CT postponement rates, female sex can be a risk factor for COV-FA-related CT postponement and advanced age is a factor that could predict increased TCBT.

**Summary points**

- We aimed to investigate how COVID-19 fear and anxiety (COV-FA) affects chemotherapy (CT) adherence in patients receiving active CT for cancer, and to identify the characteristics of patients who postponed their CT due to COV-FA, as well as the time to come back to treatment (TCBT) and the factors affecting TCBT in these patients.
- The records of 3661 patients with CT appointments during the 60-day periods preceding and following the date when the first COVID-19 case was seen in Turkey were reviewed retrospectively.
- COV-FA-related CT postponement rates were calculated before and after telemedicine.
- The CT postponement rates before and after the first COVID-19 case were 11.6 and 14.2%, respectively (p = 0.017).
- The rate of COV-FA-related CT postponement after telemedicine was lower than that before (4.6 vs 17.4%, p = 0.012).
- The median TCBT of the COV-FA group was 47 days (range 19–72). Advanced age (≥60 years) was found to be the independent factor that was predictive of TCBT (hazard ratio = 0.291, p = 0.043).
